# In silico identification of miRNAs and their target genes and analysis of gene co-expression network in saffron (*Crocus sativus *L.*) *stigma

**Published:** 2016-12

**Authors:** Zahra Zinati, Roohollah Shamloo-Dashtpagerdi, Ali Behpouri

**Affiliations:** 1 Agroecology Department, College of Agriculture and Natural Resources of Darab, Shiraz University, Iran; 2 Crop Production and Plant Breeding Department, College of Agriculture, Shiraz University, Iran

**Keywords:** *Crocus sativus*, EST sequences analysis, Co-expression network

## Abstract

As an aromatic and colorful plant of substantive taste, saffron (*Crocus sativus *L.) owes such properties of matter to growing class of the secondary metabolites derived from the carotenoids, apocarotenoids. Regarding the critical role of microRNAs in secondary metabolic synthesis and the limited number of identified miRNAs in *C. sativus*, on the other hand, one may see the point how the characterization of miRNAs along with the corresponding target genes in *C. sativus *might expand our perspectives on the roles of miRNAs in carotenoid/apocarotenoid biosynthetic pathway. A computational analysis was used to identify miRNAs and their targets using EST (Expressed Sequence Tag) library from mature saffron stigmas. Then, a gene co- expression network was constructed to identify genes which are potentially involved in carotenoid/apocarotenoid biosynthetic pathways. EST analysis led to the identification of two putative miRNAs (miR414 and miR837-5p) along with the corresponding stem- looped precursors. To our knowledge, this is the first report on miR414 and miR837-5p in *C. sativus*. Co-expression network analysis indicated that miR414 and miR837-5p may play roles in *C. sativus *metabolic pathways and led to identification of candidate genes including six transcription factors and one protein kinase probably involved in carotenoid/apocarotenoid biosynthetic pathway. Presence of transcription factors, miRNAs and protein kinase in the network indicated multiple layers of regulation in saffron stigma. The candidate genes from this study may help unraveling regulatory networks underlying the carotenoid/apocarotenoid biosynthesis in saffron and designing metabolic engineering for enhanced secondary metabolites.

## INTRODUCTION

Saffron, the desiccated stigma of *Crocus sativus *L., contains volatile and non- volatile compounds. Saffron is an important plant because of its color, taste and aroma which are resulted from accumulation of the apocarotenoids such as crocin, picrocrocin and safranal, respectively [1]. In addition, these compounds have a broad range of pharmacological effects [2, 3]. Biosynthesis and regulation of apocarotenoids is a complicated process that occurs during the growth of saffron stigma. Apocarotenoid biosynthesis occurs mainly in the context of stigma color [4]. Transcriptome analysis of saffron stigmas is a prerequisite to provide a comprehensive view of the molecular basis of carotenoid/apocarotenoid biosynthesis and corresponding regulatory networks, the gynoecium biology, and the genomic organization [5, 6]. Post-transcriptional mechanisms, such as RNA silencing, have been found to play regulatory roles in secondary metabolic synthesis [7-9]. MicroRNAs (miRNAs), recognized as vital negative post-transcriptional regulators, are 19~24 nucleotides non-coding small RNAs which can direct transcriptional repression of target genes through mRNA cleavage or translational inhibition [10-14]. Guleria et al. 2012 predicted three miRNAs of *C. sativus*, csa-miR1, csa-miR2 and csa-miR3 by using in silico methods of EST analysis [15]. In addition, predicted targets for respective miRNAs were reported to play roles in regulation of plant growth, disease resistance, senescence, stress responses, mRNA export, protein synthesis and post-translational modifications [15]. Identification and characterization of miRNAs are important, especially for metabolic engineering in crop plants. However, little is known about regulatory roles of miRNAs in saffron stigma. Mature miRNAs are highly evolutionarily conserved and homologs of known miRNAs can be identified among plant species; this has the advantage of being able to use the comparative analysis as an effective means of determination of conserved miRNAs [16]. ESTs analysis can be considered as a valuable approach for the exploration of pre- miRNAs, particularly in plant species with unavailable genome sequences. This approach has been widely performed to determine miRNAs in a growing number of plant species [17-21]. In this work, an EST library was used to discover the miRNAs expressed in Crocus stigma. Afterwards, the regulatory functions of these miRNAs were predicted by searching for potential target genes. Finally, network modeling was used to investigate the roles of putative miRNAs in carotenoid/apocarotenoid biosynthesis in Crocus stigma.

## MATERIALS AND METHODS

**Data source: **One Publicly available 5´-EST library of saffron stigma (*Crocus sativus *L., Iridaceae) containing 6202 ESTs was retrieved from NCBI (http://www.ncbi.nlm.nih.gov/). This includes the EST sequences generated by D'Agostino et al. (2007) [5]. Moreover, all currently available mature miRNAs (include 35828 sequences from 223 species) were downloaded from miRBase (http://www.mirbase.org/) [22].

**EST sequence processing and assembly: **The library was screened for vector contamination, sequence length and complexity using EGassembler webserver (http://egassembler.hgc.jp) [23]. The vector, sequences having similarity to plastids (chloroplast, mitochondrial) and repetitive sequences were masked and trimmed (less than 100 bp or having greater than 4% ambiguous bases) sequences were discarded [23, 24] and then, the remaining high quality ESTs were clustered and assembled into unigenes consisting of contigs and singletons using EGassembler webserver with overlap percent identity cutoff >80 %.

**miRNAs prediction**: The contigs were subjected against the plant mature miRNAs sequences using C-mii, a tool for plant miRNA and target identification in plants [25], with expect values=10, to increase the hit chance for more potential sequences. The minimal folding free energy index (MFEI) value of more than 0.6 is used to recognize miRNAs from other non-coding RNAs. Contig sequences having maximum 4 mis- matches were subjected to similarity searches against the non-coding RNA database 10 (Rfam 10) with removed miRNAs, and the UniProtKB/Swiss-Prot protein databases with C-mii BLAST option (E-value=10-10). All sequences with hit(s) were excluded from further analysis. miRNAs are different from other RNAs due to the distinguished property of their precursor sequences to form a secondary hairpin structure [26, 27]. Therefore, the putative miRNAs were further analyzed for being able to fold into appropriate stem–loop structures with UNAFold software embedded in C-mii. The parameters were adjusted as folding temperature (25 ºC), maximum bulges/interior loop size (30), and all others with defaults values.

**miRNA-targeted genes** **prediction and gene ontology: **The putative target genes of *C. sativus *predicted miRNAs were identified using C-mii software, with default parameters (no more than one mismatch at positions 1-9, no mismatches at positions 10 and 11 and no more than two consecutive mismatches). The mature miRNA sequences were queried against unigenes obtained from analysis of EST library of saffron stigma and unigenes of *Arabidopsis thaliana *(DFCI Gene Index (AGI), version 15) to search for putative target mRNAs. Gene ontology (GO) annotation of identified target genes was performed using the DAVID Bioinformatics Resources 6.7 [28, 29] and KEGG pathway database [30].

**Gene co-expression network: **The identified miRNA-targeted genes were networked using GeneMANIA based on automatically selected weighting method [31]. It provides a number of co-expressed genes relevant to target genes to make the regulatory networks more complete.

Identification of genes encoding transcription factor and protein kinases:

Homology-based search against the plant transcription factor database (PlnTFDB) [32] was performed in order to identify genes encoding transcription factor in the network. Also, homology-based search against the Kinase Sequence Database (KSD) [33] was performed in order to identify genes encoding protein kinases in the network.

## RESULTS AND DISCUSSION

Pre-processing of 6202 EST sequences derived from saffron stigma resulted in 6020 high quality sequences. Assembling pre-processed ESTs resulted in 912 unigenes consisting of 609 contigs and 303 singletons, in which 5717 (94.93%) of ESTs fell into the contigs. The average contigs length was 508bp. The number of ESTs ranged from 2 to 555 in the contigs indicating that the genes have been expressed at different levels. 303 ESTs remained as singletons that probably represented the genes that are expressed at low level.

**Figure 1:**
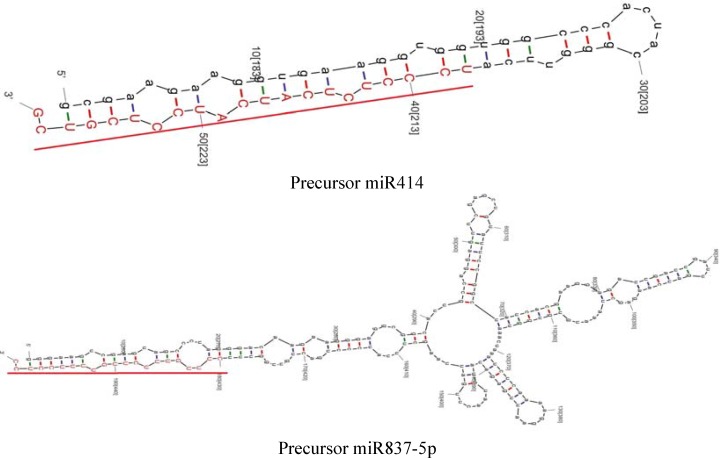
Predicted secondary structure of in silico identified precursor miRNAs in *C. sativus *L. The mature miRNAs sequences are underlined and highlighted in red

The alignment of total contigs obtained from EST analysis with known miRNAs from the miRNA Registry (miRBase) identified two contig sequences (contig 65 and contig 441) possessing the features so as to be considered as a putative miRNA and showed complementarities with two conserved plant mature miRNAs (ppt-miR414 and aly-miR837-5p, respectively). The number of ESTs in contig 65 and contig 441 was 8 and 2, respectively. The lengths of pre-miRNA414 and pre-miRNA837 were 58bp and 200bp, respectively. Both in silico predicted miRNAs were located at 3' ends of their respective pre-miRNAs. Minimum free energy (MFE), minimum free energy index (MFEI) values and some features of miRNAs were presented in Table 1. The secondary structures of the identified miRNAs were presented in Fig. 1. miRNAs are key components of large gene regulatory networks. Identification of miRNAs and miRNA target genes has a great significance to plant genetic improvement. In saffron, identification and characterization of miRNAs through genetic screening and direct cloning is difficult due to the non-availability of genomic sequence data and the tissue and time specific expressions of some miRNAs [34]. Computational approaches as powerful tools have simplified the identification and characterization of potential miRNAs. In doing so, two putative miRNAs including miR837-5p and miR414 along with the corresponding stem-looped precursors were computationally identified among the contigs, that have broadened our understanding of miRNAs which may play crucial regulatory roles in flavor and color biogenesis, the gynoecium biology, and the genomic organization. Unlike highly conserved mature miRNAs, pre-miRNAs are known to be species specific. Because it shows direct evidence of the of miRNA gene expression, the identification of pre-miRNAs in any organism is of fundamental importance [16].

According to Xie et al. (2010), the miR414 existed across the plant kingdom several million years ago prior to the divergence of monocots and dicots. It can therefore be found in almost all plant species [35]. According to research by Guleria and Yadav (2011) on miR414 expression in *Stevia rebaudiana*, miR414 primarily targets transcriptional regulators which participate in vital plant functions including growth, development, physiological and morphological changes, metabolism and defense responses. Hence, miR414 seems to play an essential role in the regulation of growth and development of plant [36]. A number of researchers have reported the important role of miR414 targets in post-transcriptional modifications, including SNF2 transcriptional regulator, high mobility group proteins, pentatricopeptide repeat- containing proteins, C2H2 zinc finger proteins, F-Box family proteins, DNA store keepers and RNA recognition motifs [37-40]. In addition, miR414 was reported to be a drought stress-associated miRNA in *Physcomitrella patens*, indicating that it may play important roles in drought stress tolerance in *P. patens*. It has been shown that mir414 targets mainly are involved in transportation of protein or sugar. Mir414 targets including group 3 late embryogenesis abundant (LEA) proteins and sucrose transporter showed overexpression in response to drought stress [41]. To our awareness, there has been little discussion about the processes regulated by miR837-5p in plants. Hua and coworkers conducted a survey and showed that miR837-5p in Arabidopsis, targets genes related to iron deficiency [42].

**Table 1:** Some features of predicted miRNAs from EST analysis of *C.* *sativus*

**Known mature miRNA**	**ppt-miR414**	**aly-miR837-5p**
Input sequence name	**Contig65**	**Contig441**
Number of mismatches between miRNA and miRNA*	3	4
Number of bulges	2	0
Bulge size	1	0
Number of flanked bases	2	8
Precursor start	174	251
Precursor stop	231	450
Primary miRNA MFE(kcal/mol)	-187.405	-293.481
Precursor miRNA MFE(kcal/mol)	-28.295	-66.852
Precursor miRNA MFEI(kcal/mol)	-0.832	-0.777
Number of homolog mismatches	4	4
Known mature miRNA sequence	5':UCAUCCUCAUCAUCCUCGUCC:3'	5':CAUUGUUUCUUGUUUUUUUCA:3'
Predicted miRNA sequence	5':UCCCUCUCAUCAUCCUCGUCG:3'	5':CUUUGUUUCUUGUUUUCCUCC:3'

The maximum asymmetry of miRNA/miRNA

* duplex was 4 for prediction of a novel miRNA

Target prediction is the first step to infer the roles of the putative miRNAs. So, miRNA targets were predicted. Each of putative miRNAs targeted 5 loci. Target prediction of newly identified miRNAs is presented in Table 2. Substantiated by UPE (unpaired energy), the most putative target genes for miR414 and miR837-5p were AT4G38480 and AT5G04670, respectively (Table 2). When mature miRNA sequences were used as queries to search for putative targets in unigenes obtained from analysis of saffron stigma EST library, only one EST (accession: EX143431.1) was considered to be miR414 target. The targets of miRNAs were also investigated in respect to their gene ontology. The putative miRNAs targets presented diverse biological process, molecular functions and cellular components (Table 2). Predicting the targets of miRNAs can provide informative clues about potential functions of miRNA and the miRNA regulatory network in saffron stigma. In our EST library one singletone (accession: EX143431.1) is found to be targeted by miR414. Subjecting this singletone to a tBLASTn search against nucleotide collection (nr/nt) database, showed it probably was beta carbonic anhydrase 5. Annotation of target protein suggested that this protein is involved in carbonate dehydratase activity. Search against KEGG pathway database was performed to elucidate the biochemical pathway in which this gene is involved.

**Table 2:** The list of potential targets predicted for the putative miRNAs from *C. sativus *and their corresponding gene ontology

**miRNA**	**Target genes**	**Description**	UPE*	**Inhibition ** **mechanism **	**Gene Ontology**		
					**process**	**function**	**component**
**miR414**	gb|EX143431.1| EX143431	Beta carbonic anhydrase 5	20.468	Translation	Carbonate dehydratas e activity	Metal ion binding	Chloroplas, plastid
	AT2G13700.1	Transposable element gene	9.032	Cleavage			
	AT4G06613.1	Transposable element gene	20.119	Cleavage			
	AT3G29783.1	Transposable element gene	6.812	Cleavage			
	AT4G38480.1	Transducin/WD40	16.214	Cleavage		Nucleotide	CUL4 RING
	(F20M13_40)	repeat-like superfamily protein				binding	ubiquitin ligase complex
**miR837-5p**	AT2G15670.1	SEC14 cytosolic factor family protein / phosphoglyceride transfer family protein	14.724	Cleavage			Integral component of membrane
	AT5G04670.1 (T1E3_30)	Enhancer of polycomb-like protein	16.168	Cleavage	Regulation of		Nucleus(Picco lo NuA4
					transcription		histone acetyltransfera se complex)
	AT5G48790.1	Domain of unknown function (DUF1995)	15.571	Cleavage	photosynt hesis, light reaction,		Chloroplast, plastid
					regulation of protein dephospho		
					rylation		
	AT1G52565.1	unknown protein	15.838	Cleavage			Endomembran e system
	AT3G42770.1	F-box/RNI-like/FBD- like domains-		Cleavage			

Based on the results, it might be involved in nitrogen metabolism. No target gene was found for miR837-5p in unigenes obtained from analysis of saffron stigma EST library. However, 5 target genes for miR837-5p were predicted in Arabidopsis. Gene ontology analysis showed that some of the predicted target genes were involved in transcription regulation, photosynthesis, light reaction, and regulation of protein dephosphorylation.

A study by Eisen and colleagues suggested that co-expressed genes were functionally related. Sets of co-expressed genes that may be associated with target genes can be elucidated by gene co-expression network building. Co-expression analyses can lead to characterize genes of unknown function [43]. In doing so, the gene co- expression network was constructed, using potential target genes of predicted miR414 and miR837-5P. The list of predicted co-expressed genes is presented in Table 3.

**Figure 2:**
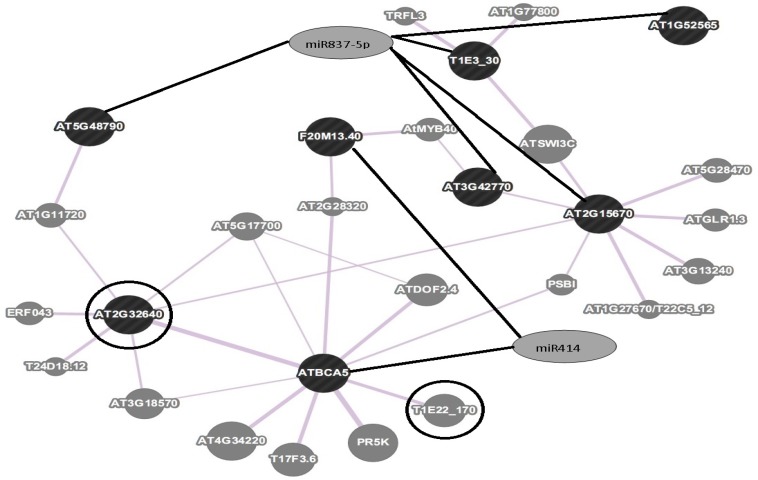
A graphical view of co-expression network of potential target genes. Network was constructed using GeneMANIA based on automatically selected weighting method. Network includes predicted miRNAs, miRNA-target genes and co-expressed genes. Relations between miRNAs and target genes are shown as black edges, whereas relations between target genes and co-expressed genes are depicted by gray edges. Lycopene beta/epsilon cyclase protein (AT2G32640) was directly or indirectly connected to almost all of the target genes of predicted miR414 and miR837-5P. The co-expression network also unraveled the relationship between beta carbonic anhydrase 5 (a target of miR414) and alpha-1,2- glucosyltransferase (T1E22_170). Genes involved in carotenoid/apocarotenoid biosynthesis (AT2G32640 and T1E22_170) are highlighted with a circle

The network uncovered the relationships between the target genes and the co- expressed genes. Lycopene beta/epsilon cyclase protein (AT2G32640) was shown to be a part of the co-expression network that included 28 relationships (Fig. 2) and was directly or indirectly connected to almost all of the target genes. Earlier reports have also shown increased expression of *LCYβ *and *LCYε *in stigma leading to increased production of secondary metabolites [44]. Notably, lycopene beta/epsilon cyclase protein within the predicted network was connected to the carotenoid biosynthesis. Additionally, beta carbonic anhydrase 5, target of miR414, had a relationship with alpha-1,2-glucosyltransferase (Fig. 2). Glucosyltransferases are enzymes that catalyze the transfer of a sugar molecule to a speci fi c acceptor, thereby forming a glucosidic bond. Many secondary metabolites of higher plants, including important fl avour compounds, are glucosylated [45]. Carotenoid-derived metabolites (apocarotenoids) constitute a growing class of secondary metabolites that are found as glucosides in fruits and other plant parts [46]. In *C. sativus*, crocetin and 3-Hydroxy-*β*-cyclocitral are transformed to crocin and picrocrocin respectively by enzymatic activities of UDP- glucosyltransferases [6]. To date the gene encoding alpha-1,2-glucosyltransferase has not been reported in crocus. Therefore, kinetic analysis and substrate specificity of this enzyme from crocus may help elucidate the role it plays in apocarotenoid biosynthetic pathway. Identification and characterization of glycosyltransferase genes from saffron stigma could help explain the accumulation of a specific glycosylated metabolite [47]. It is noteworthy that miR414 and miR837-P targeted the genes that co-expressed with gene such as lycopene beta/epsilon cyclase protein (AT2G32640) which was reported to be involved in carotenoid biosynthesis. This finding suggested the significant role of predicted miRNAs in carotenoid biosynthetic process (Fig. 2).

**Table 3:** The list of co-expressed genes in the co-expression network

**Symbol**	**Tair ID**	**Description**	**Pathway**
AT1G52565 ATBCA5	AT1G52565 AT4G33580	unknown protein beta carbonic anhydrase 5	Nitrogen
			metabolism
AT2G32640	AT2G32640	Lycopene beta/epsilon cyclase protein	
T1E3_30	AT5G04670	Enhancer of polycomb-like transcription factor protein	
AT2G15670	AT2G15670	BEST Arabidopsis thaliana protein match is: SEC14 cytosolic factor family protein / phosphoglyceride transfer family protein	
F20M13.40	AT4G38480	Transducin/WD40 repeat-like superfamily protein	
AT3G42770	AT3G42770	F-box/RNI-like/FBD-like domains-containing protein	
AT5G48790	AT5G48790	Domain of unknown function (DUF1995)	
AT4G34220	AT4G34220	Leucine-rich repeat protein kinase family protein	
ATSWI3C	AT1G21700	SWITCH/sucrose nonfermenting 3C	
PR5K	AT5G38280	PR5-like receptor kinase	
T17F3.6	AT1G69910	Protein kinase superfamily protein	
T1E22_170	AT5G02410	alpha-1,2-glucosyltransferase	N-Glycan biosynthesis
ATDOF2.4	AT2G37590	DNA binding with one finger 2.4	
AT3G18570	AT3G18570	Oleosin family protein	
AT5G17700	AT5G17700	MATE efflux family protein	
AT3G13240	AT3G13240	unknown protein	
AT1G11720	AT1G11720	starch synthase 3	
AT5G28470	AT5G28470	Major facilitator superfamily protein	
AtMYB40	AT5G14340	myb domain protein 40	
ERF043	AT4G32800	Integrase-type DNA-binding superfamily protein	
ATGLR1.3	AT5G48410	glutamate receptor 1.3	
PSBI	ATCG0008	photosystem II reaction center protein I	
T22C5_12	AT1G27670	unknown protein; BEST Arabidopsis thaliana protein match is:	
		unknown protein (TAIR:AT1G75360.1)	
AT2G28320	AT2G28320	Pleckstrin homology (PH) and lipid-binding START domains-	
T24D18.12	AT1G16020	Protein of unknown function (DUF1712)	
TRFL3	AT1G17460	TRF-like 3	
AT1G77800	AT1G77800	PHD finger family protein	

Despite the significant progress concerning carotenoid/apocarotenoid pathway [1, 48], enzymes, intermediates [6] and the network regulating the carotenoid/apocarotenoid biosynthesis still remain elusive. Based on the co-expression network presented here, it can be suggested that these putative miRNAs will provide a knowledge base about regulatory network of carotenoid/apocarotenoid biosynthesis. Identification of transcription factor and protein kinase genes in this network may provide a new insight into the regulatory mechanism of carotenoid/apocarotenoid biosynthesis. Six unigenes including *Arabidopsis thaliana *switching protein 3C, Dof- type zinc finger domain-containing protein, AtMYB40, AP2 domain-containing transcription factor TINY, TRF-LIKE 3 and PHD finger family protein showed similarity to transcription factors belonging to various families (Table 4).

Transcription factors are known to play key roles in plant growth, development and response to various environmental stimuli. They are also reported to be involved in regulation of secondary metabolite biosynthesis [6]. Presence of the identified transcription factors in the co-expression network supports previous researches into investigation expression of regulatory carotenoid pathway genes. Recent evidence suggest that several members of Myb family have regulatory roles in different secondary metabolic pathways [49-51]. Gargouri et al. demonstrated that *PHD7 *was positively correlated with zeaxanthin epoxidase (*ZEO*), enzyme involved in carotenoid biosynthesis in *Chlamydomonas *[52]. A survey conducted by Baba et al. (2015) showed that zinc finger protein DOF5.4 and zinc finger protein DOF2.4 were expressed in saffron stigma. Since little is known about the transcriptional regulators controling the expression of structural genes in Crocus carotenoid/apocarotenoid biosynthetic pathway, the transcription factors identified from this survey are good candidates to experimentally validate their roles in carotenoid/apocarotenoid biosynthetic pathway and flower development [6]. Also, protein kinases are involved in proteinsphosphorylation. Phosphorylation and dephosphorylation as the major general mechanism controlling proteins [53] regulates diverse cellular functions in eukaryotes such as cell division, cell differentiation, signal transduction, etc [6]. One unigene showed similarity to leucine-rich repeat transmembrane protein kinase (AT4G34220) belonging to family98.

**Table 4:** The list of co-expressed genes encoding transcription factors in the co-expression network

**Symbol**	**Tair ID**	**Description**	**Family**
ATSWI3C	AT1G21700	ATSWI3C (Arabidopsis thaliana switching protein 3C); DNA binding	MYB-related
ATDOF2.4	AT2G37590	Dof-type zinc finger domain-containing protein	C2C2-Dof
AtMYB40	AT5G14340	AtMYB40 (myb domain protein 40); DNA binding / transcription factor	MYB
ERF043	AT4G32800	AP2 domain-containing transcription factor TINY, putative	AP2-EREBP
TRFL3	AT1G17460	TRFL3 (TRF-LIKE 3); DNA binding / transcription factor	MYB-related
AT1G77800	AT1G77800	PHD finger family protein	PHD

Taken together, these findings indicated the presence of miRNAs in regulatory pathway of carotenoid/apocarotenoid biosynthesis in saffron stigma. Target prediction and co-expression network led to the inference regarding the roles of the predicted miRNAs. The identified miRNAs and their target and co-expressed genes including transcription factors and protein kinase in *C. sativus *may help unraveling regulatory networks underlying the carotenoid/apocarotenoid biosynthesis and designing metabolic engineering for enhanced secondary metabolites. Due to the existence of false positive results in bioinformatics prediction, putative miRNA discovered through this approach need to be further verified with laboratory experiments. This survey provides an overview of the existence and expression of miRNAs in saffron sigma for future research.
